# *Lactobacillus plantarum* MH-301 as an effective adjuvant to isotretinoin in the treatment of acne vulgaris: a randomized and open-label trail

**DOI:** 10.3389/fmed.2023.1340068

**Published:** 2024-01-09

**Authors:** Lili Liang, Xinyue Qi, Xiaoke Jiang, Tingtao Chen, Lina Dong

**Affiliations:** ^1^Department of Dermatology, Shanxi Provincial People’s Hospital (Fifth Hospital) of Shanxi Medical University, Taiyuan, China; ^2^Institute of Life Science, Nanchang University, Nanchang, China; ^3^National Engineering Research Center for Bioengineering Drugs and the Technologies, Institution of Translational Medicine, Jiangxi Medical College, Nanchang University, Nanchang, China; ^4^School of Pharmacy, Jiangxi Medical College, Nanchang University, Nanchang, China; ^5^Core Laboratory, Shanxi Provincial People’s Hospital (Fifth Hospital) of Shanxi Medical University, Taiyuan, China

**Keywords:** acne vulgaris, *Lactobacillus plantarum*, isotretinoin, efficacy, acne

## Abstract

**Introduction:**

Acne vulgaris is a common chronic inflammatory skin disease originating in the sebaceous gland units of the skin follicles. Isotretinoin is presently the primary choice for the treatment of acne vulgaris. However, it could induce several adverse reactions like diarrhea, cheilitis, headache, elevated triglyceride levels and risk of inflammatory bowel disease and depression. Hence, it is imperative to seek an alternative therapy.

**Methods:**

One hundred five patients were randomly divided into 3 groups, and received a baseline treatment of oral doxycycline for the initial 4 weeks. Group I received isotretinoin oral for 12 weeks; Group P received oral *Lactobacillus plantarum* MH-301 treatment for 12 weeks; Group IP received combined treatment with oral probiotics and oral isotretinoin for 12 weeks. The number of skin lesions was recorded at 0, 4, 8, and 12 weeks during the treatment to compare the efficacy of each intervention, and skin and fecal samples were collected from patients at 12 weeks for high-throughput sequencing to explore the microbiota differences between various groups.

**Results:**

Our results revealed that the combination of *L. plantarum* MH-301 with isotretinoin significantly reduced the number of skin lesions in patients compared to using *L. plantarum* MH-301 and isotretinoin alone (*p* < 0.001). Additionally, skin microbiome High-throughput analysis indicated the restorative effects of *L. plantarum* MH-301 on skin microbial diversity while also observing a reduction in the main microbiota of skin lesions, *Propionibacterium* and *Corynebacterium*. Meanwhile, gut microbiome High-throughput analysis showed that it could regulate disorders of the intestinal microbiota and increased the abundance of probiotics such as *Lactobacillus*, *Bifidobacterium*, *Coprococcus* and *Bacteroides* genera.

**Conclusion:**

In conclusion, *L. plantarum* MH-301 could be used in combination with isotretinoin for optimal results in the treatment of acne vulgaris. The research conducted provides theoretical and data support for the adjuvant effect of *L. plantarum* in the treatment of acne vulgaris.

**Clinical Trial Registration:**

[ClinicalTrials.gov], identifier (ChiCTR2200063499).

## 1 Introduction

Acne vulgaris, characterized by disturbance of skin microbiota, represents a persistent inflammatory dermatological condition originating in the pilosebaceous units of the skin, and afflicts roughly 10% of the worldwide populace in any age group, especially in adolescents (1, 2). If left untreated, acne commonly causes scarring and exerts a profound impact on multiple dimensions of a patient’s life, including vocational, social, and academic domains (3). Currently, there are several treatment options available for acne vulgaris, including topical antibiotics, phototherapy and oral retinoids such as isotretinoin (4). Isotretinoin, which is believed to act on all proposed mechanisms of acne development, represents the optimal choice for long-term management of acne vulgaris (1). However, it can cause numerous common dose-dependent adverse effects, covering diarrhea, cheilitis, headache, elevated triglyceride levels and liver enzymes (5–8). Previously studies have reported some cases association between isotretinoin and inflammatory bowel disease (IBD), mood disorders, and suicidal ideation (9–11). Hence, an imperative exists to investigate refined therapeutic approaches aimed at enhancing the effectiveness of isotretinoin for the treatment of acne vulgaris while simultaneously mitigating its associated side effects.

The human intestine harbors a multitude of diverse microbial communities, which assume a pivotal role in the preservation of human health. Cumulative evidences link gastrointestinal (GI) dysbiosis to skin disorders (12, 13). For instance, a recent study demonstrated a notable decrease in the abundance of *Bifidobacterium*, *Actinobacteria*, *Coprobacillus*, *Butyricicoccus* and *Lactobacillus* species, concomitant with an increased prevalence of *Proteobacteria* within individuals afflicted by acne vulgaris (14). The disruption of gut microbiota influence pathophysiology of acne via metabolic inflammation, immunologic derangement and impaired cell proliferation (15). Moreover, during the period of isotretinoin treatment, isotretinoin may exert an inhibitory response that impacts luminal bacterial stimulation by impairing the innate immune response, potentially resulting in an excessive immune reaction, subsequently contributing to gastrointestinal inflammation and metabolic dysregulation (16). A mice model had demonstrated that isotretinoin induces notable damage to the intestinal mucosa, resulting in the formation of ulcers within the gastrointestinal tract which in turn break the gut microbial homeostasis (17). Conversely, it may be possible to moderate isotretinoin and treat acne by regulating intestinal microbiota. Deng et al. suggested that transplantation of modified gut microbiota mitigated systemic inflammation and ameliorated the acne phenotype in their rat model (18). A clinical investigation conducted with a cohort of participants aged between 18 and 30 years verified a reduction in inflammatory lesions by 38.6% compared to a placebo group. Additionally, a selective decrease in triacylglycerols was observed in acne-afflicted patients following 12 weeks of daily consumption of fermented milk enriched with 200 mg of lactoferrin (19). Therefore, regulating intestinal microbiota could be an innovative therapeutic target for acne treatment and isotretinoin improvement.

In light of the recognized influence of intestinal dysbiosis in the pathogenesis of inflammatory skin conditions, the use of probiotic supplementation emerges as a promising alternative or adjuvant therapeutic strategy for the management of acne. Defined by the beneficial living microorganisms, probiotics are thought to maintain health and treat disorders like diarrhea, depression, and diabetes via improving intestinal microbiota, enhancing intestinal barrier, regulating immune system, and modulating metabolic process (20). In a preliminary pilot study, the administration of *Lactobacillus rhamnosus* SP1 over a 12 weeks period led to the suppression of IGF-1 signaling, ultimately resulting in reduced keratinocyte proliferation and mitigated sebaceous gland hyperplasia (21). In an investigation involving obese diabetic mice, the administration of *Bifidobacterium* species demonstrated the inhibition of endotoxemia induced by a high-fat diet and suppressed systemic inflammation through a mechanism dependent on GLP-2. Elevated levels of glucagon-like peptide 2 (GLP-2), a proglucagon-derived peptide with intestinotrophic properties, were associated with improved tight junction integrity and reduced intestinal permeability. These effects may potentially serve to mitigate the collateral effects of isotretinoin (22). Therefore, the incorporation of probiotics as an adjuvant therapy for acne may offer a less aggressive treatment option.

In this present research, we recruited 105 patients with acne vulgaris and selected *L. plantarum* MH-301 as the oral supplement of isotretinoin to assess the therapeutic effect on acne vulgaris. In addition, changes of gut and skin microbiota were evaluated by high-throughput sequencing. The research endeavors to furnish empirical evidence supporting the incorporation of probiotics as an adjunctive treatment for acne in conjunction with isotretinoin.

## 2 Materials and methods

### 2.1 Participants recruitment and enrolment

From September 27, 2021 to November 19, 2022, the study was constructed in the form of a single-center, open-label, parallel groups study. Patient recruitment was conducted at the Shanxi Provincial People’s Hospital, where all patients had comprehensive clinical records available. Prior to their involvement in the experiment, all patients signed written informed consent. The following eligibility criteria were applied: patients of acne vulgaris aged between 18 and 30 had facial lesions between 20 and 50 and achieve grade III to IV according to the Chinese Acne Treatment Guidelines with more than 10 inflammatory acne lesions on their face. Patients who met the criteria had no history of systemic disease. The exclusion criteria were as follows: people who smoke, are pregnant, breastfeeding; patients with a history of allergy to drugs or probiotics; individuals had taken oral isotretinoin within the last 6 months or taken oral antibiotics or probiotics in the last 1 month for acne; individuals with a history any other form of topical or systemic anti-acne treatment in the last 1 month; patients had concurrent dermatological conditions that might influence the assessment of acne such as folliculitis or rosacea.

This trial was registered with the China Clinical Trials Center and assigned the registration number ChiCTR2200063499. This research received ethical approval from the Ethics Committee of Shanxi Provincial People’s Hospital.

### 2.2 Group design and treatment

In the present study (23), it was assumed that the expected treatment effect of control group would result in a 10.00% reduction in inflammatory skin lesions, while the intervention group would reach a 56.67% reduction (power = 0.8, *α* = 0.05) Accounting for a 20% attrition rate, each group was intended to enroll 19 participants at least. Thus, a total of 105 individuals were recruited in this study.

Patients (*n* = 105) meeting the eligibility criteria received a baseline treatment of oral doxycycline (20–30 mg/kg/day) for the initial 4 weeks. Following this phase, they were randomized into three groups: Group I (*n* = 35) receiving oral isotretinoin 50 mg/kg/day for 12 weeks; Group P (*n* = 35) receiving oral *L. plantarum* MH-301 (CGMCC No. 18618, Harbin Meihua Biotechnology Co., Ltd., Harbin, Heilongjiang, China) treatment at a dosage of 2 g/day (10^9^ CFU/g) for 12 weeks; Group IP (*n* = 35) receiving combined treatment with oral probiotics (2 g/day, 10^9^ CFU/g) and oral isotretinoin (50 mg/kg/day) for 12 weeks. The probiotic used in this experiment was a mixture of *L. plantarum* MH-301 and maltodextrin with 2 × 10^9^ colony-forming units per packet of live bacteria. The probiotic preparation stored in a refrigerator at 4°C. Skin samples collections and lesion counts were performed at four different time points: before treatment, at 4 weeks, 8 weeks, and 12 weeks during the treatment. Feces samples were collected at 12 weeks of the treatment. Feces samples were collected using 15 mL sterile tubes and stored at −80°C refrigerator for high-throughput sequencing analysis.

### 2.3 Clinical outcome assessments

In this study, the lesion counting method was chosen as the clinical assessment approach for acne vulgaris. Facial photographs were captured under standardized conditions at 0 week, 4 weeks, 8 weeks, and 12 weeks of treatment, respectively, and skin lesion counting was conducted by the same dermatologist. This counting included both non-inflammatory lesions, such as open and closed comedones, and inflammatory lesions, including papules, and pustules according to the Chinese Acne Treatment Guidelines. Importantly, the dermatologist conducting the lesion counting was kept unaware of the patient groupings, ensuring an unbiased assessment.

### 2.4 Skin and feces sample collection

To detect the difference of skin microbiota in each group, patient skin samples were collected at 4 weeks, 8 weeks and 12 weeks of treatment. After moistening a sterile cotton swab with physiological saline, the swab was gently used to scrape the surface of skin lesion. Following the sampling, the tip of the cotton swab was cut off and placed into a sterile EP tube. The tube was sealed tightly and stored in a −80°C freezer pending subsequent sequencing. Moreover, for the fecal microbiota, feces samples were collected at 12 weeks of treatment in centrifuge tubes and stored in a refrigerator at −80°C awaiting sequencing analysis.

### 2.5 DNA extraction and16S rRNA high-throughput sequencing

Total genomic DNA was extracted from skin and feces samples of patients using the TIANamp Bacteria DNA Kit (QIAGEN) in accordance with the manufacturer’s instructions and from glass fiber filters. The genomic DNA concentration and quality extracted were evaluated using a spectrophotometer (NanoDrop, Thermo Fisher Scientific, Inc., United States). The DNA extracted underwent 16S sequencing at Shanghai Personal Biotechnology Company Ltd. The primer sets 338F (5′-ACTCCTACGGGAGGCAGCA-3′) and 806R (5′-CGGACTACHVGGGTWTCTAAT-3′) were used to amplify the hypervariable V3V4 region of the 16S rRNA gene in the skin samples, while primer sets 520F (5′-AYTGGGYDTAAAGNG-3′) and 802R (5′-TACNVGGGTATCTAATCC-3′) were used to amplify the V4 hypervariable region in feces samples. The PCR-amplified products were double-end sequenced utilizing the Illumina MiSeq platform. FLASH was used to merge overlapped reads, and sequence analysis was carried out using UPARSE software package. Reads with quality scores lower than 20, ambiguous bases, and improper primers were discarded before clustering, and chimeras were detected and removed during the process. ASV/OTU signature sequences were acquired through the application of the DADA2 approach, followed by processing via Quantitative Insights into Microbial Ecology (QIIME). The taxonomic categorisation was carried out utilizing the Greengenes version 13.8 database (19), where the percentage of total bacteria in every sample was placed into dissimilar classification tiers (Phylum, Class, Order, Family, and Genera). The samples collected from Groups M, I, P, and IP underwent analysis for α-diversity, β-diversity, and species differences.

The α-diversity, including Chao1 and Observed species indices (represented richness), Shannon and Simpson indices (represented diversity), and Goods-coverage index (represented coverage), were calculated and visualized by QIIME2 and R software (v 3.6.3, R Foundation for Statistical Computing). The β-diversity, which is based on Jaccard distances and unweighted UniFrac distances, was demonstrated by PCoA to show the dissimilarity of species composition, and was calculated and visualized using QIIME2 and R software. The distributions of skin and feces microbiota at both phylum and genus levels were analyzed and visualized using QIIME2, and the results were shown as histograms.

### 2.6 Statistical analysis

Prism version 9.5.0 (GraphPad Prism, San Diego, CA, United States) or SPSS 27.0.1 were used for data analysis. Continuous variables of normality distributions were shown as mean ± standard deviation (SD), qualitative data are expressed as rates, and one-way ANOVA with Tukey’s multiple comparison test was employed to assess the variations between groups. Covariates were selected based on gender, age and total number of skin lesions prior to treatment. Descriptive statistics were used to describe the characteristics of the recruited participants and were assessed using chi-square tests and one-way ANOVA to assess baseline comparability of age and sex between the different groups, respectively. Two-way ANOVA was used to assess differences in skin lesion counts between groups of patients at 4 weeks, 8 weeks and 12 weeks of treatment. Statistical significance was set at ^*^p < 0.05, ^**^p < 0.01, and ^***^p < 0.001.

## 3 Results

### 3.1 Patient baseline characteristics

**Figure 1:**
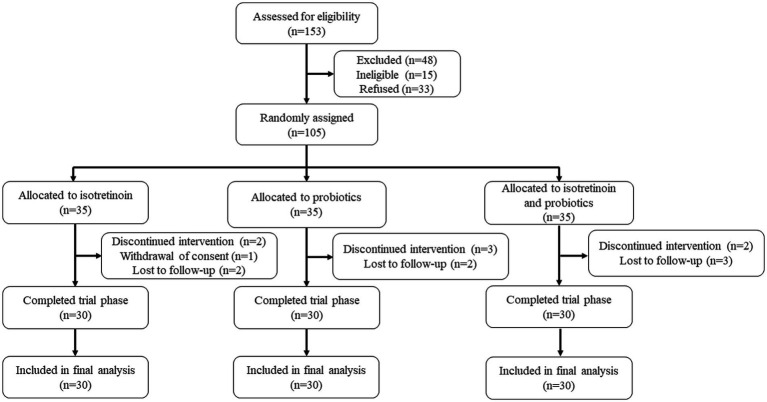
Flowchart of the trial.

In order to assess the adjuvant efficacy of probiotics in conjunction with isotretinoin for the treatment of acne vulgaris, a total of 153 individuals were recruited to assess for eligibility, of whom 105 individuals were initially screened for eligibility and subsequently enrolled into the study (Figure 1). They were randomly assigned, with 35 participants allocated to each of the oral isotretinoin group (Group I), oral *L. plantarum* MH-301 group (Group P), and oral combination of isotretinoin and *L. plantarum* MH-301 group (Group IP). There was no significant difference in the patients’ gender (*p* > 0.05) and age (I vs. P vs. IP = 22.68 vs. 21.72 vs. 22.44, *p* > 0.05). Regarding the total skin lesion count of baseline, no significant difference was found among the three groups (I vs. P vs. IP = 61.70 vs. 59.50 vs. 61.03, *p* > 0.05). Thus, based on clinical records, the recruited patients across the three groups were homogeneous (Table 1). During the course of the study, one participant in Group I withdrew consent and 2 patients received discontinued intervention, while 2 participants were lost to follow-up. A total of 2 and 3 participants were lost to follow-up in Groups P and IP, respectively. Totally, 3 and 2 patients were received discontinued intervention in Groups P and IP. Ninety participants successfully completed the study and were subsequently incorporated into the final analysis (Figure 1).

**Table 1:** Baseline characteristics.

	I	P	IP	*p*-value
Age (mean ± SD) (years)	22.68 ± 2.78	21.72 ± 3.54	22.44 ± 3.59	0.749
**Gender, *n* (%)**				
Female	18 (51.4)	20 (57.1)	21 (60.0)	0.763
Male	17 (48.6)	15 (42.9)	14 (40.0)	
Total skin lesion count (mean ± SD)	61.70 ± 10.00	59.50 ± 9.74	61.03 ± 6.46	0.357

### 3.2 *Lactobacillus plantarum* MH-301 significantly enhanced the efficacy of isotretinoin in acne vulgaris

To evaluate whether *L. plantarum* MH-301 can improve acne vulgaris and enhance the therapeutic effect of isotretinoin on acne vulgaris, we administered oral isotretinoin (Group I), oral *L. plantarum* MH-301 (Group P), and an oral combination of isotretinoin and *L. plantarum* MH-301 (Group IP) to three groups, respectively, and monitored the number of skin lesions in patients at weeks 0, 4, 8 and 12 of treatment. The result of each intervention approach was measured by calculating the average number of changes in skin lesions before and after treatment within the same group, and then calculated the percentage reduction in skin lesions. Clinical results showed that comparing to the amount of skin lesions of baseline, the severity of acne vulgaris significantly reduced in patients following oral administration of isotretinoin and *L. plantarum* MH-301 (Supplementary Figure S1).

**Figure 2:**
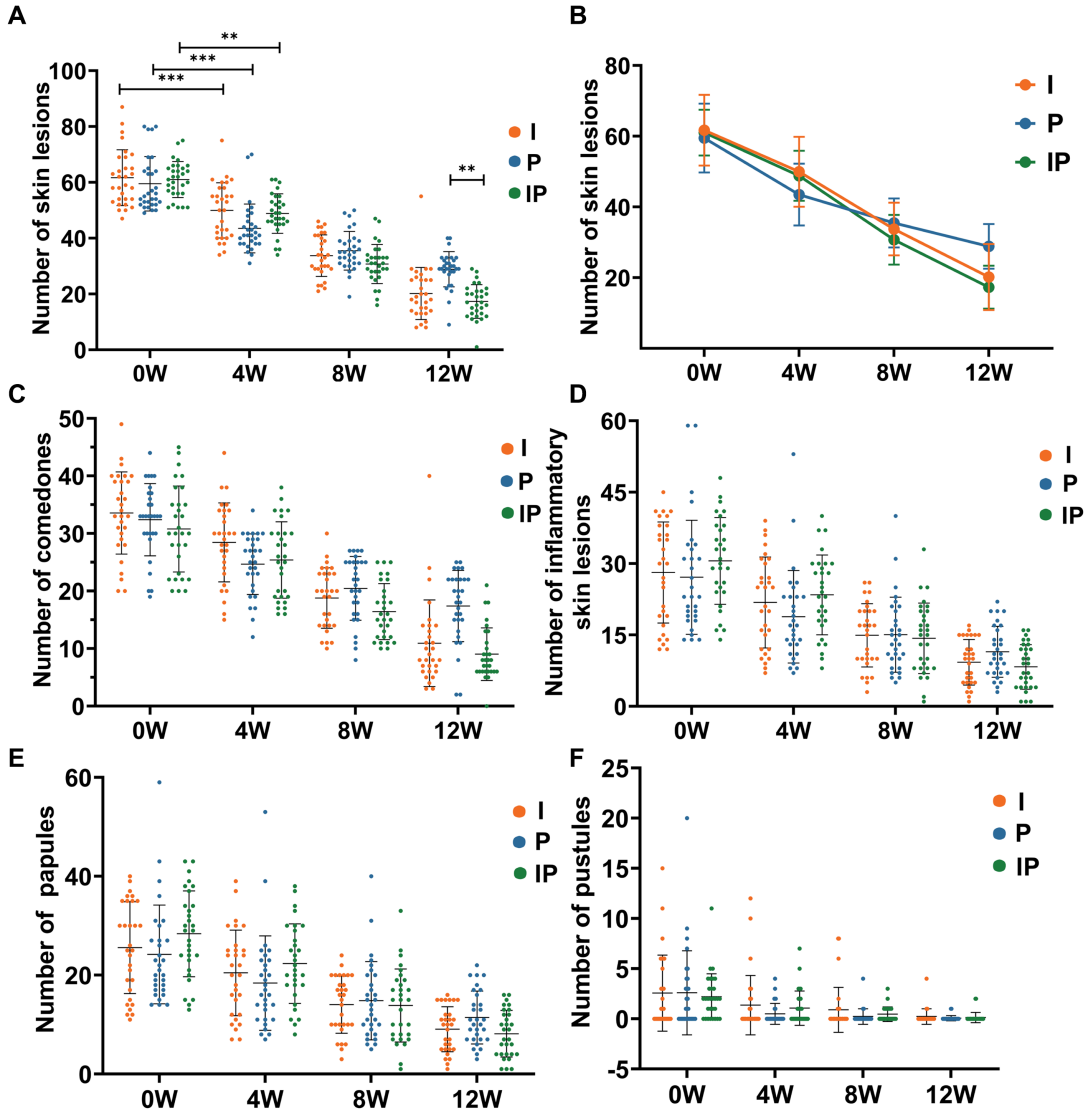
Oral isotretinoin and *L. plantarum* MH-301 reduce the number of skin lesions in patients with acne vulgaris. **(A)** Histograms of the changes in the number of skin lesions in each group during the treatment period (*n* = 30). **(B)** Line graphs of the changes in the number of skin lesions in each group during the treatment period (*n* = 30). **(C)** Changes in the number of comedones in each group during the treatment period (*n* = 30). **(D)** Changes in the total number of inflammatory skin lesions in each group during the treatment period (*n* = 30). **(E)** Changes in the total number of papules in each group during the treatment period (*n* = 30). **(F)** Changes in the total number of pustules in each group during the treatment period (*n* = 30). ^*^p < 0.05, ^**^p < 0.01, ^***^p < 0.001.

The reduction in skin lesions of each group was statistically significant as early as the fourth week of treatment, oral isotretinoin treatment (Group I) exhibited a significant average reduction in skin lesions19.02% (baseline vs. the fourth week = 61.70 vs. 49.97, *p* < 0.001). Compared to baseline, oral *L. plantarum* MH-301 (Group P) showed a reduction of 26.89% (baseline vs. the fourth week = 59.50 vs. 43.50, p < 0.001), and oral combination of isotretinoin and *L. plantarum* MH-301 group (Group IP) exhibited a reduction of 19.99% (baseline vs. the fourth week = 61.03 vs. 48.83, *p* < 0.001), and this improvement was sustained until the completion of the treatment course (Figures 2A,B and Supplementary Table S1). Overall, the amount of skin lesions in Group I was less than Group P, while group IP treatment had the largest reduction in skin lesion, especially in the 8- and 12 weeks during course (reductions of the twelfth week in Group I vs. P vs. IP = 20.20 vs. 28.87 vs. 17.33, *p* < 0.01) (Figure 1). By the twelfth week, Group I achieved a reduction of 67.26% (baseline vs. the twelfth week = 61.70 vs. 20.20, *p* < 0.001), Group P achieved a reduction of 51.48% (baseline vs. the twelfth week = 59.50 vs. 28.87, *p* < 0.001), while Group IP showed a reduction of 71.60% (baseline vs. the twelfth week = 61.03 vs. 17.33, *p* < 0.001) (Supplementary Table S1).

Then we further identify and counted non-inflammatory lesions, namely comedones and inflammatory lesions including pustules of papules. For comedones, the patients’ symptoms improved as the treatment progressed, and the effect was most significant at week 12, with Group IP having the best results comparing to the baselines (baselines vs. week 12 of Group IP = 30.80 vs. 9.03, *p* < 0.001) (Figure 2C), likewise with pimples and pustules (Figures 2D–F). Collectively, these results suggested that *L. plantarum* MH-301 have the ability to improve skin lesions of acne vulgaris significantly in short time, and *L. plantarum* MH-301 can enhance the effects of isotretinoin on acne to achieve long-term and best intervention effect.

### 3.3 *Lactobacillus plantarum* MH-301 as auxiliary to improve the dysbiosis of skin microbiota and remodeled the skin microbial taxa in acne vulgaris patients

**Figure 3:**
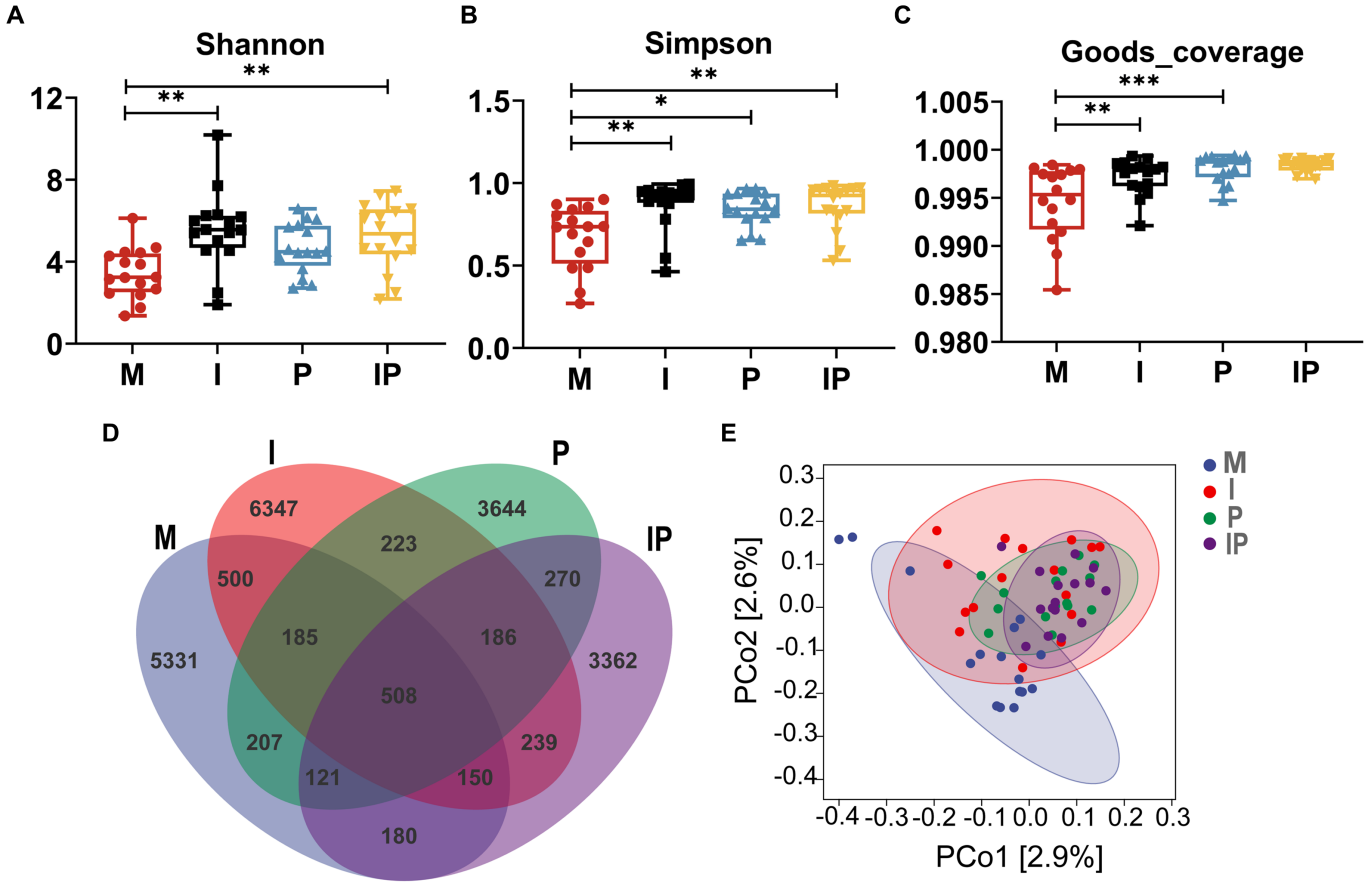
*L. plantarum* MH-301 and isotretinoin improve skin microbiota dysbiosis in patients with acne vulgaris. **(A)** Shannon index; **(B)** Simpson index; **(C)** Goods-coverage index; **(D)** Venn diagram; **(E)** principal coordinate analysis (PCoA analysis). ^*^p < 0.05, ^**^p < 0.01, ^***^p < 0.001.

Studies indicated a close association between acne vulgaris and disturbances in the skin microbiota. Therefore, we collected and sequenced microbiota from skin lesion sites of patients. A total of 64 skin samples from lesion sites were collected at week 12 of treatment, comprising 16 patients in each of the Group M, I, P, and IP. Subsequently, we subjected these samples to 16S rRNA sequencing analysis to investigate alterations in the skin microbial community. The α-diversity analysis showed that the Shannon index (*p* < 0.01) (Figure 3A), Simpson index (*p* < 0.01) (Figure 3B), and Goods-coverage index (*p* < 0.001) (Figure 3C) of Group I, P and IP increased dramatically compared with the baseline (Group M, for medicine not used), suggesting that both isotretinoin and *L. plantarum* MH-301 may be effective in restoring the diversity of skin microbiota that was disrupted by acne vulgaris. Furthermore, results from Venn diagrams (Figure 3D) revealed the presence of 508 shared Operational Taxonomic Units (OTUs) across all four groups, and the unique OTU numbers discovered in groups M, I, P, and IP were 5,331, 6,347, 3,644 and 3,362 respectively, indicating that acne vulgaris may impact the types of species that inhabited the skin and that treatment with *L. plantarum* MH-301 alone can hardly reshape this change. Additionally, principal coordinate analysis (PCoA analysis) revealed slight differences in patients between Group I, P and IP compared to Group M (Figure 3E), which further suggested that acne vulgaris affected the structure of human skin microbiota and *L. plantarum* MH-301 could remodel the skin microbial profile.

**Figure 4:**
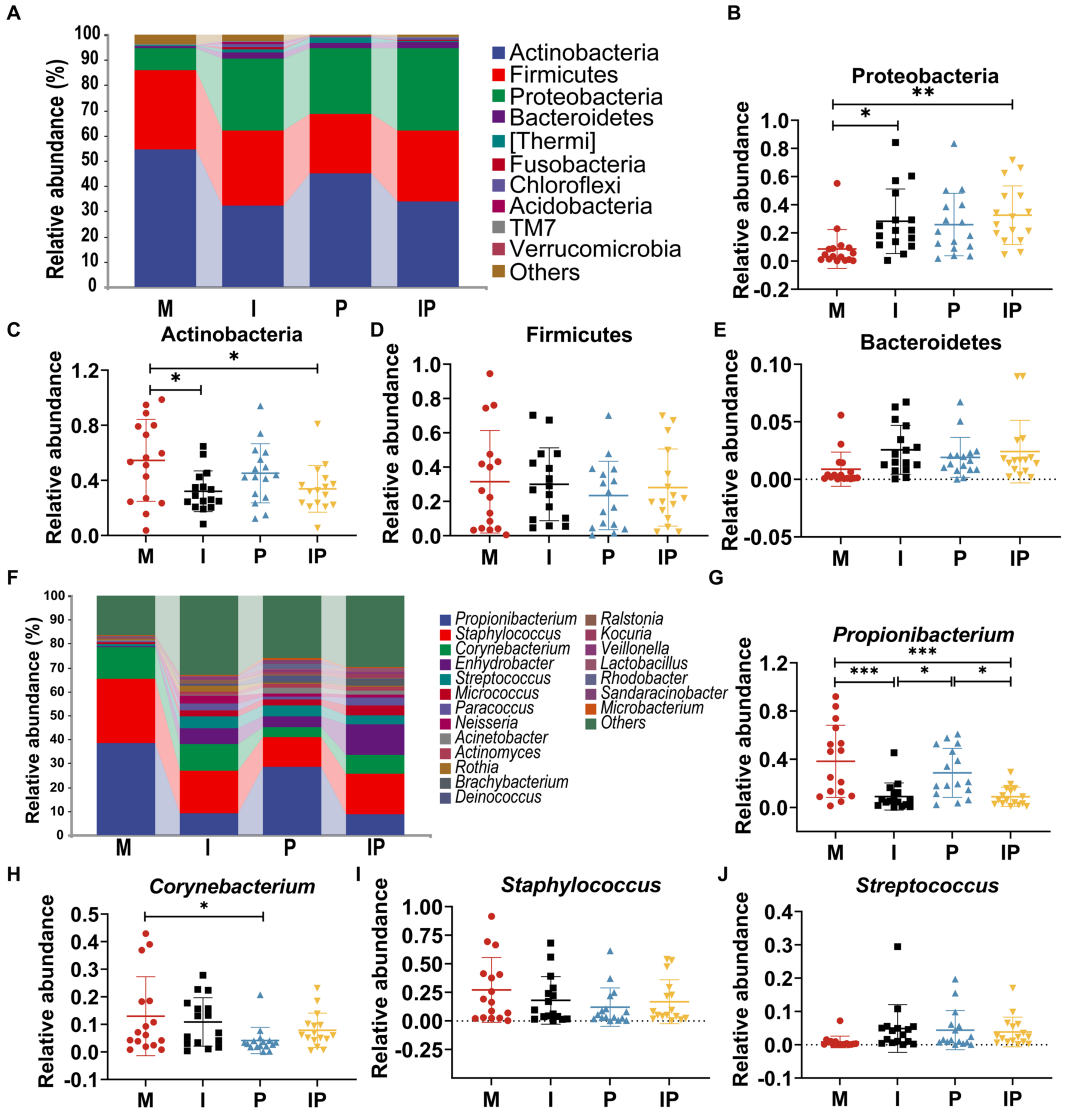
Oral isotretinoin and *L. plantarum* MH301 can restore the skin microbiota in patients with acne vulgaris. **(A)** Skin microbiota abundance analysis at the phylum level. **(B–E)** The relative abundance of Proteobacteria, Actinobacteria, Firmicutes and Bacteroidetes. **(F)** Skin microbiota abundance analysis at the genus level. **(G–J)** The relative abundance of *Propionibacterium*, *Corynebacterium*, *Staphylococcus* and *Streptococcus*. ^*^p < 0.05, ^**^p < 0.01, ^***^p < 0.001.

At the phylum level, Actinobacteria, Firmicutes, Proteobacteria and Bacteroidetes were the most prevalent populations in these treated groups, accounting for 95.62, 93.11, 96.60 and 97.06% of the total sequencing results, respectively (Figure 4A). Specifically, the relative abundance of Proteobacteria (Figure 4B) was 27.37% in oral isotretinoin group, which was significantly higher than that in baseline (Group M) (8.65%, *p* = 0.038), oral combination of isotretinoin and *L. plantarum* MH-301 group was also significantly higher than the Group M (32.63% vs. 8.65%, *p* = 0.007). In contrast, the relative abundance of Actinobacteria (Figure 4C) in Group I and IP were significantly higher than that in Group M (I vs. IP vs. M = 32.12% vs. 33.89% vs. 54.58%, *p* = 0.02, *p* = 0.04). Additionally, the abundance of Firmicutes (M vs. I vs. P vs. IP = 31.51% vs. 30.05% vs. 23.49% vs. 28.12%) and Bacteroidetes (M vs. I vs. P vs. IP = 0.88% vs. 2.57% vs. 1.91% vs. 0.34%) had no differences between groups, suggesting that they were may not affected by acne vulgaris, yet the abundance of Firmicutes could be reduced by *L. plantarum* MH-301 preparation and Bacteroidetes could be increased (Figures 4D,E).

At the genus level, we selected representative skin microbiota closely associated with acne vulgaris for further analysis (Figure 4F). The abundance of *Propionibacterium* and *Corynebacterium* were lower in the oral isotretinoin and *L. plantarum* MH-301 treatment groups alone and in the combination treatment groups (Group I, P and IP) than in the baseline (Group M). In concrete terms, the relative abundance of *Propionibacterium* (Figure 4G) in Group I, P and IP was 9.02, 28.67, 8.83% respectively, while in Group M was 38.26% (M vs. I = 38.26% vs. 9.02%, *p* = 0.0004, M vs. IP = 38.26% vs. 8.83%, *p* = 0.004). The relative abundance of *Corynebacterium* (Figure 4H) in Group I, P and IP was 10.89, 4.14 and 7.87%, while in Group M was 12.95% (M vs. *p* = 12.95% vs. 4.14%, *p* = 0.04). These results suggest that *L. plantarum* MH-301 could partially reverse the change trend of the skin microbiome. Additionally, the content of *Staphylococcus* and *Streptococcus* were not influenced by acne vulgaris as their relative abundance were similar among Group M, I, P, and IP (*Staphylococcus* M vs. I vs. P vs. IP = 27.19% vs. 18.04% vs. 12.11% vs. 16.85%, *Streptococcus* M vs. I vs. P vs. IP = 0.85% vs. 4.90% vs. 4.41% vs. 3.85%), but after treatment, the abundance of *Staphylococcus* decreased and the abundance of *Streptococcus* increased (Figures 4I,J). Collectively, these results suggested that *L. plantarum* MH-301 could improve the dysbiosis of skin microbiota, and remodel the skin microbial taxa in acne vulgaris patients, particularly in decreasing *Propionibacterium*, *Corynebacterium* and *Staphylococcus* and increasing *Streptococcus*.

### 3.4 *Lactobacillus plantarum* MH-301 reversed intestinal dysbiosis and restored intestinal microbiota diversity

**Figure 5:**
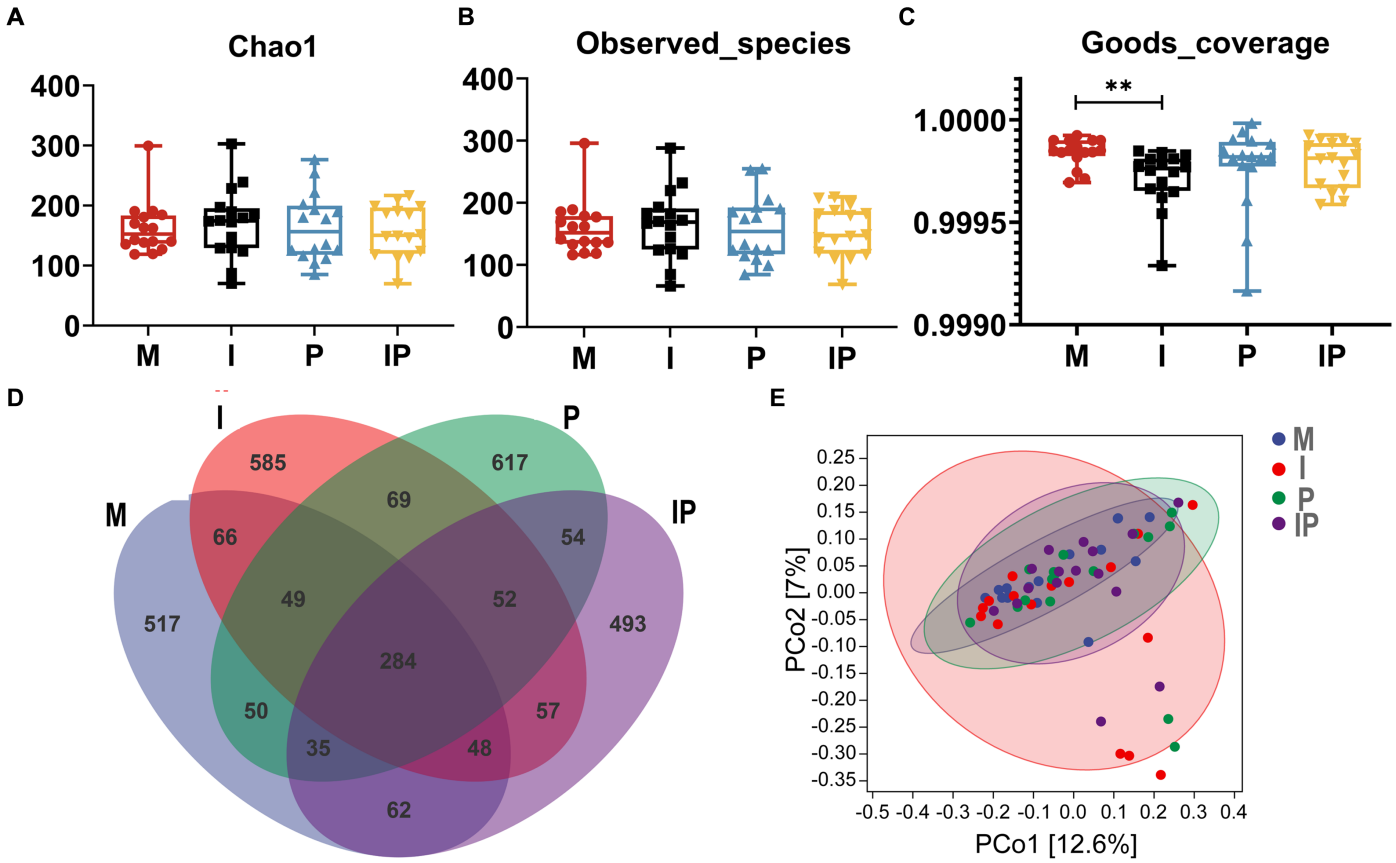
*L. plantarum* MH-301 and isotretinoin improve gut microbiota dysbiosis in patients with acne vulgaris. **(A)** Chao1 index; **(B)** Observed-species index; **(C)** Goods-coverage index; **(D)** Venn diagram; **(E)** Principal coordinate analysis (PCoA analysis). ^*^p < 0.05, ^**^p < 0.01, ^***^p < 0.001.

Previous studies have indicated a possible association between acne vulgaris and the gut microbiota. We prescribed patients oral isotretinoin and *L. plantarum* MH-301 and collected their feces for 16S rRNA sequencing analysis to assess alternations in the gut microbiota of the patients. A total of 64 feces samples were collected (16 from the Group M, 16 from the Group I, 16 from the Group P, and 16 from the Group IP). It turns out that, for α-diversity, there were differences among the four groups in terms of Chao1, Observed-species, and Goods-coverage, but these differences did not reach statistical significance (Figures 5A–C). In particular, the Chao 1 and Observed-species in oral *L. plantarum* MH-301 group (Group P and IP) were marginally greater than that of in baseline (Group M), while *L. plantarum* MH-301 could restore the diversity of gut microbiota that was disrupted by acne vulgaris. Venn diagram results (Figure 5D) indicated that there were 284 common operational taxonomic units (OTUs) across all four groups, with unique OTUs numbering 517, 585, 617, and 493 for the Group M, I, P and IP, which further shown that acne vulgaris might affect the gut microbiological composition of patients, but only *L. plantarum* MH-301 could hardly remodel this change. Moreover, principal coordinate analysis (PCoA analysis) did not differ significantly between Group M, I, P and IP (Figure 5E).

**Figure 6:**
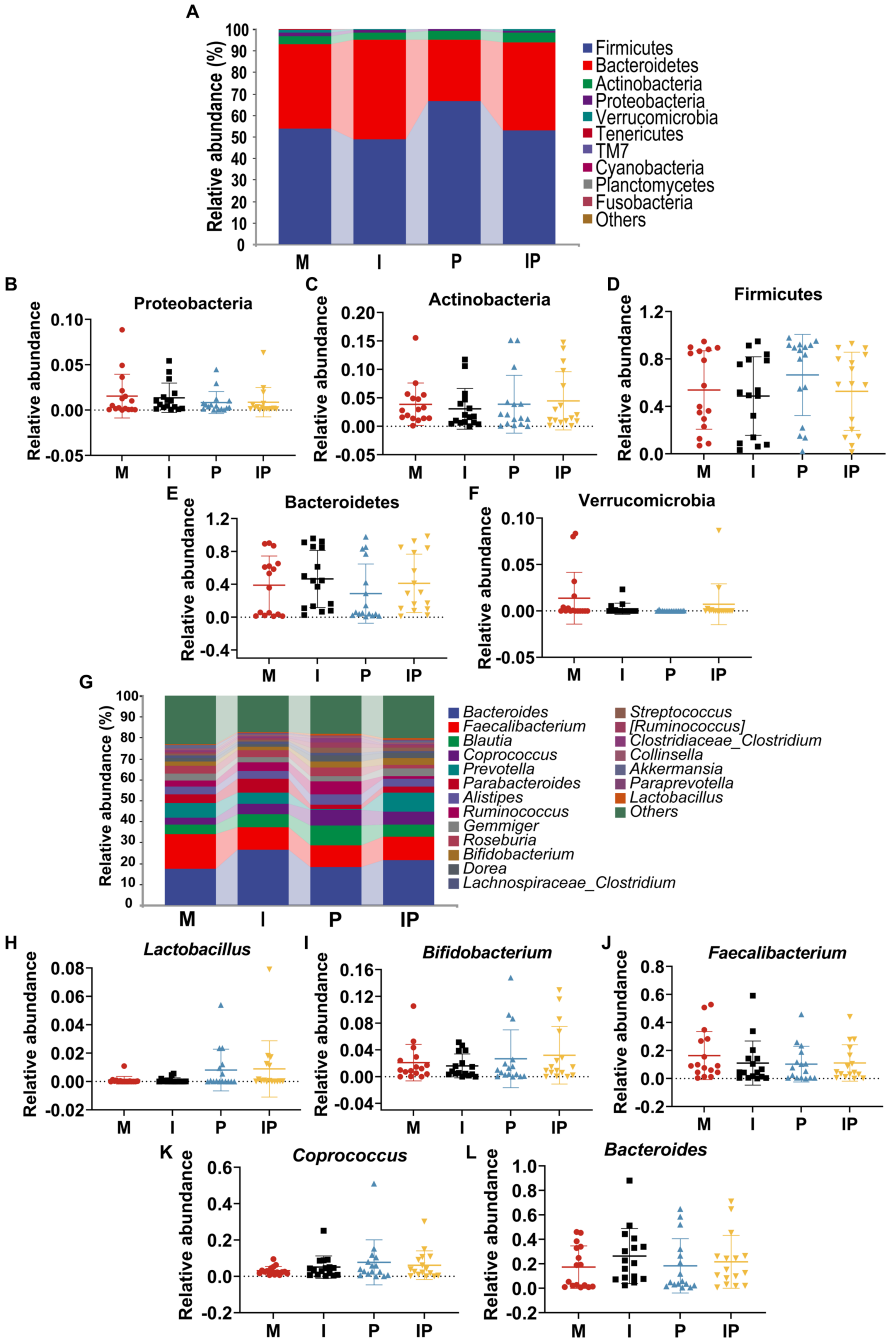
Oral isotretinoin and *L. plantarum* MH301 can restore the gut microbiota in patients with acne vulgaris. **(A)** Gut microbiota abundance analysis at the phylum level. **(B–F)** The relative abundance of Proteobacteria, Actinobacteria, Firmicutes, Bacteroidetes and Verrucomicrobia. **(G)** Gut microbiota abundance analysis at the genus level. **(H–L)** The relative abundance of *Lactobacillus*, *Bifidobacterium*, *Faecalibacterium*, *Coprococcus* and *Bacteroides*.

At the phylum level, Firmicutes, Bacteroidetes, Actinobacteria, Proteobacteria and Verrucomicrobia were the most common microbiota in the patients’ gut, and they accounted for 98.10, 99.67, 99.88 and 99.26% of the sequencing results for each group, respectively (Figure 6A). Remarkably, after oral administration of *L. plantarum* MH-301, the abundance of Actinobacteria in Group I and IP were slightly higher than that in Group M (I vs. IP vs. M = 3.89 and 4.48% vs. 3.87%) (Figure 6C). In contrast, the relative abundance of Proteobacteria and Verrucomicrobia in oral *L. plantarum* MH-301 group (Group P and IP) (0.84 and 0.87%, 0.01 and 0.72%) were lower than that in baseline (Group M) (1.54, 1.37%) (Figures 6B,F). Additionally, *L. plantarum* MH-301 treatment showed opposite changes in the abundance of Firmicutes and Bacteroidetes, but the same changes in Group I and IP (Figures 6D,E).

At the genus level (Figure 6G), the fecal microbiota of patients in the probiotic treatment groups including Group P and IP were richer in *Lactobacillus* (0.81 and 0.85%), *Bifidobacterium* (2.68 and 3.20%), *Coprococcus* (7.68 and 6.12%), and *Bacteroides* (18.36 and 21.68%) than that in baseline (Group M) (0.09, 2.09, 3.09 and 17.31%) (Figures 6H,I,K,L). On the contrary, the abundance of *Faecalibacterium* reduced in Group P and IP. These results suggested that *L. plantarum* MH-301 had the potential to counteract certain alterations within the gut microbiota and restore gut homeostasis (Figure 6J). Collectively, these results suggested that *L. plantarum* MH-301 could help regulate and improve gut dysbiosis caused by acne vulgaris, thereby remodel intestinal microbiota diversity, especially in decreasing *Faecalibacterium* and increasing *Lactobacillus*, *Bifidobacterium*, *Coprococcus* and *Bacteroides*.

## 4 Discussion

Acne vulgaris, an inflammatory skin disease, has its origins in the sebaceous glandular units of the skin follicles (23). The primary clinical treatment for acne vulgaris is oral isotretinoin. However, given the notable side effects associated with isotretinoin, there is an urgent need to explore and develop optimized therapeutic strategies (24). Recent research has provided mounting evidence that probiotics, particularly *Lactobacillus*, hold promise as a potential treatment for acne vulgaris. These probiotics might alleviate disruptions in gut microbiota and mitigate varying levels of depression often observed in acne patients (25). Therefore, we proposed a novel therapeutic approach, combining oral probiotics with isotretinoin, to evaluate the potential efficacy of probiotic-assisted isotretinoin in the treatment of acne vulgaris.

In this study, we enrolled 105 patients with acne vulgaris and divided them equally into isotretinoin treatment group (Group I), *L. plantarum* MH-301 treatment group (Group P), and isotretinoin and *L. plantarum* MH-301 combination treatment group (Group IP). The aim of this study was to assess the adjunctive role of *L. plantarum* MH-301 in conjunction with isotretinoin for the treatment of acne vulgaris. Our results indicated that the combination of *L. plantarum* MH-301 with isotretinoin treatment led to a significant reduction in the number of skin lesions. This reduction was observed for both non-inflammatory lesions, such as comedones, and inflammatory lesions, including pustules and papules, over the 4, 8, and 12 weeks treatment period. Furthermore, high-throughput sequencing analysis of skin and fecal samples demonstrated the ability of the combination therapy to improve dysbiosis in both skin and gut microbiota among the treated patients.

Skin lesions are the predominant symptom in patients with acne vulgaris, primarily resulting from hyperkeratinization of hair follicles and inflammation triggered by the colonization of *Staphylococcus* and *Propionibacterium* (26). Notably, Podrini et al. (27) discovered that *L. plantarum* possessed the ability to inhibit lipid production, reduced anti-inflammatory markers, and diminished the presence of *Propionibacterium* and *Staphylococcus* in human primary sebocytes. In our study, we observed a significant reduction in the number of skin lesions, including comedones and inflammatory lesions like papules and pustules, by the fourth week of oral *L. plantarum* MH-301 treatment (Group P and IP) (Figure 2). It’s worth noting that the most substantial reduction in skin lesions occurred in Group P, suggesting that the therapeutic effect of probiotics was more effective than that of the traditional drug isotretinoin at week 4, likely owing to the anti-inflammatory and antibacterial properties of *L. plantarum* MH-301 (1). However, after 8 weeks of treatment, patients who received daily oral combination of *L. plantarum* MH-301 and isotretinoin exhibited more pronounced overall improvements in acne symptoms than those receiving either component alone. This trend was even more evident after 12 weeks of treatment. These findings corroborated well with previous results that single strain of *L. plantarum* improved acne lesion count and grade in patients with acne vulgaris (28, 29). This evidence suggested that both *L. plantarum* MH-301 and isotretinoin hold promise in the treatment of acne vulgaris, with *L. plantarum* MH-301 assisting isotretinoin in achieving better therapeutic results through its anti-inflammatory and antibacterial properties.

The dysbiosis of skin microbiota has been found to be closely related to the development of various skin diseases, like atopic dermatitis and acne vulgaris (30). Previous research has indicated that the severity of acne vulgaris is inversely correlated with the diversity of skin microbiota in affected patients (31), findings that align with our own observations. In this study, the oral *L. plantarum* MH-301 group (Group P and IP) showed a noteworthy increase in α-diversity, as measured by the Shannon index, Simpson index, and Goods-coverage index. Additionally, there was a modest change in β-diversity as revealed by principal coordinates analysis (PCoA) compared to the control group (Group M) (Figure 3). These findings collectively indicate that the reduced microbial diversity on the skin of acne patients may be attributed to the overgrowth of pathogenic microorganisms (31). Importantly, our results demonstrate that *L. plantarum* MH-301 can effectively restore the diminished diversity of skin microbiota associated with acne vulgaris (31).

The surface of the skin is home to a diverse array of commensal microorganisms, which include genera such as *Propionibacterium*, *Corynebacterium*, and *Staphylococcus*, forming a complex and vital barrier. Disruptions and reductions in these beneficial microorganisms can weaken this barrier, leading to various skin disorders. In our study, we observed that as the treatment progressed, by the twelfth week, there was a notable increase in the abundance of *Propionibacterium*, *Corynebacterium*, and *Staphylococcus* genera. Importantly, treatment with *L. plantarum* MH-301 partially reversed these microbial shifts, contributing to the restoration of the skin barrier integrity (Figure 4).

The concept of the gut-skin axis has gained prominence due to the recognized close relationship between gut homeostasis and skin health. Research has unveiled that gastrointestinal dysbiosis can exert a direct or indirect influence on the host’s skin immunity, contributing to various skin disorders, including acne vulgaris (15, 32). Furthermore, several studies have demonstrated that a high diversity of gut microbiota typically results in a more balanced gut environment (33). In our study, we observed an elevation in the chao1 index of intestinal α-diversity and the observed species index in the treatment group, while β-diversity showed only modest alterations (Figure 5). These findings suggest that the probiotic preparation may play a role in maintaining gut homeostasis, which in turn can contribute to the improvement of acne vulgaris symptoms (34).

Numerous studies have uncovered the role of gut microbial composition in contributing to inflammatory dermatoses (35, 36). Moreover, the intake of probiotics, such as *Lactobacillus* and *Bifidobacterium*, has been shown to reduce dermatoses (37). Notably, research has demonstrated that fecal microbiota transplantation (FMT) from healthy mice to atopic dermatitis (AD) mice leads to alterations in the gut microbiota, including a significant increase in *Lactobacillus* in the treatment group and a reduction in AD skin lesions (38), findings consistent with our own results. Furthermore, AD patients often exhibit a reduction in *Coprococcus* in their gut, as this genus is capable of producing anti-inflammatory short-chain fatty acids (SCFA) (38). In our study, sequencing of fecal samples from patients also showed revealed an increase in the levels of *Coprococcus* as the treatment progressed. When compared to Group M, the relative abundances of *Lactobacillus*, *Bifidobacterium*, *Coprococcus*, and *Bacteroides* genera all increased in the probiotic-treated groups (P and IP), while *Faecalibacterium* genera decreased (Figure 6). These findings collectively suggest that oral *L. plantarum* MH-301 may regulate the intestinal microbiota through its anti-inflammatory action in the gut. This regulation, in turn, contributes to the improvement of acne vulgaris.

The trial was conducted with a rigorous design, encompassing stringent inclusion and exclusion criteria, as well as comprehensive clinical judgment criteria. To mitigate potential confounding factors, all assessments were performed by the same dermatologist. Additionally, we delved into the microbiological aspects, investigating changes in both skin and gut microbiota. While our study has shed light on the potential role of *L. plantarum* MH-301 in restoring gut and skin microbiota, there are several limitations that merit consideration. In acknowledgment of potential limitations pertaining to sample size adequacy, our study confronts challenges stemming from the protracted duration of dermatological treatment and inherent difficulties associated with patient follow-up. Despite these constraints, a comprehensive examination of statistical methodologies has informed our determination that a cohort of 35 individuals in each group aligns with the calculated minimum sample size, thus affording a statistically robust foundation for the reflection of our study outcomes. Also, for skin diseases, controlling diet is also crucial for treatment. But, unlike animals, it’s unrealistic to control diet because of the long-term treatment of skin disease. Moreover, we lacked controls from the normal population in fecal 16S rRNA sequencing and more clinical indicators, which are also a deficiency of this study. In addition, we analyzed the clinical symptoms and microbiological changes in the skin and gut of patients with acne vulgaris. However, a more in-depth exploration of the potential mechanisms through animal models of inflammatory dermatoses and advanced molecular techniques is warranted. Specifically, elucidating the intricate interplay between the microbiota-gut-skin axis and the biological functions of *L. plantarum* MH-301 is essential. These efforts are pivotal for the future clinical application of probiotics.

## 5 Conclusion

**Figure 7:**
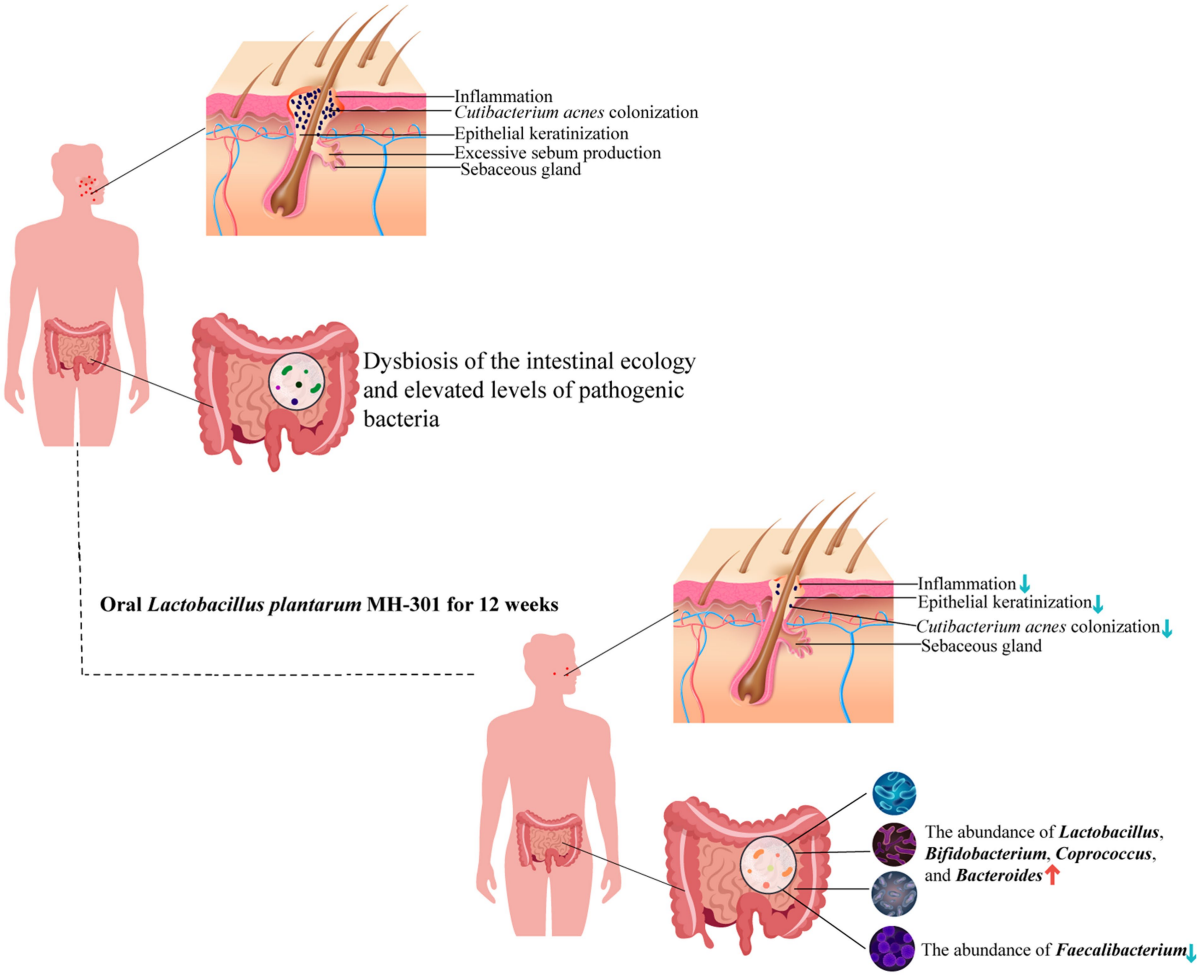
Schematic representation of the potential mechanisms of *L. plantarum* MH-301 to ameliorate acne vulgaris. *L. plantarum* MH-301 restored skin microbiota, reduced the colonization of *Cutibacterium acnes* and hyperkeratinization of the skin, thereby reducing skin inflammation, and also improved intestinal dysbiosis.

In conclusion, the findings from the present study highlighted the synergistic potential of *L. plantarum* MH-301 when combined with oral isotretinoin, significantly enhancing the therapeutic efficacy of isotretinoin in mitigating the symptoms of acne vulgaris and reducing the number of skin lesions in patients (Figure 7). Moreover, our results demonstrated that oral *L. plantarum* MH-301 is effective in reinstating the homeostasis of both skin and gut microbiota. Through multilevel analysis, we offered insights into how alterations in intestinal microbiota diversity and changes in marker bacteria may underpin the observed effects of *L. plantarum* MH-301. This study provided a solid theoretical foundation for the use of *L. plantarum* in combination with isotretinoin for the treatment of acne vulgaris. Nonetheless, further clinical trials are warranted to validate these mechanistic insights.

## Data availability statement

The datasets presented in this study can be found in online repositories. The names of the repository/repositories and accession number(s) can be found in the article/Supplementary material.

## Ethics statement

The studies involving humans were approved by the Ethics Committee of Shanxi Provincial People’s Hospital. The studies were conducted in accordance with the local legislation and institutional requirements. The participants provided their written informed consent to participate in this study. Written informed consent was obtained from the individual(s) for the publication of any potentially identifiable images or data included in this article.

## Author contributions

LL: Conceptualization, Funding acquisition, Project administration, Supervision, Writing – original draft. XQ: Data curation, Investigation, Methodology, Writing – review & editing. XJ: Resources, Software, Validation, Writing – original draft. TC: Formal analysis, Writing – original draft. LD: Conceptualization, Visualization, Writing – original draft.
