# Supplementary material for: Lactobacillus plantarum MH-301 as an effective adjuvant to isotretinoin in the treatment of acne vulgaris: a randomized and open-label trail
Source: Front Med (Lausanne). 2024 Jan 9;10:1340068. doi: 10.3389/fmed.2023.1340068 (PMC10803606; doi:10.3389/fmed.2023.1340068)
Supplement: Supplementary file 1 [file Table_1.DOCX]

**Table S1.** Difference in skin lesions before and after treatment in each group.

|  | **I** | **P** | **IP** |
| --- | --- | --- | --- |
| Skin lesion count (Baseline) (Mean ± SD) | 61.70 ± 10.00 | 59.50 ± 9.74 | 61.03 ± 6.46 |
| Skin lesion count after 4 weeks treatment (Mean ± SD) | 49.97 ± 9.89 | 43.50 ± 8.73 | 48.83 ± 7.08 |
| Mean difference (Mean ± SD) | 11.73 ± 1.50 | 16.00 ± 4.32 | 12.20 ± 2.90 |
| Percent decrease in mean skin lesion count | 19.02% | 26.89% | 19.99% |
| p-value | < 0.001 | < 0.001 | < 0.001 |
| Skin lesion count after 12 weeks treatment (Mean ± SD) | 20.20 ± 9.33 | 28.87 ± 6.30 | 17.33 ± 6.05 |
| Mean difference (Mean ± SD) | 41.50 ± 3.61 | 30.63 ± 7.43 | 43.70 ± 2.26 |
| Percent decrease in mean skin lesion count | 67.26% | 51.48% | 71.60% |
| p-value | < 0.001 | < 0.001 | < 0.001 |
