# Supplementary material for: Lactobacillus plantarum MH-301 as an effective adjuvant to isotretinoin in the treatment of acne vulgaris: a randomized and open-label trail
Source: Front Med (Lausanne). 2024 Jan 9;10:1340068. doi: 10.3389/fmed.2023.1340068 (PMC10803606; doi:10.3389/fmed.2023.1340068)
Supplement: Supplementary file 2 [file Data_Sheet_1.docx]

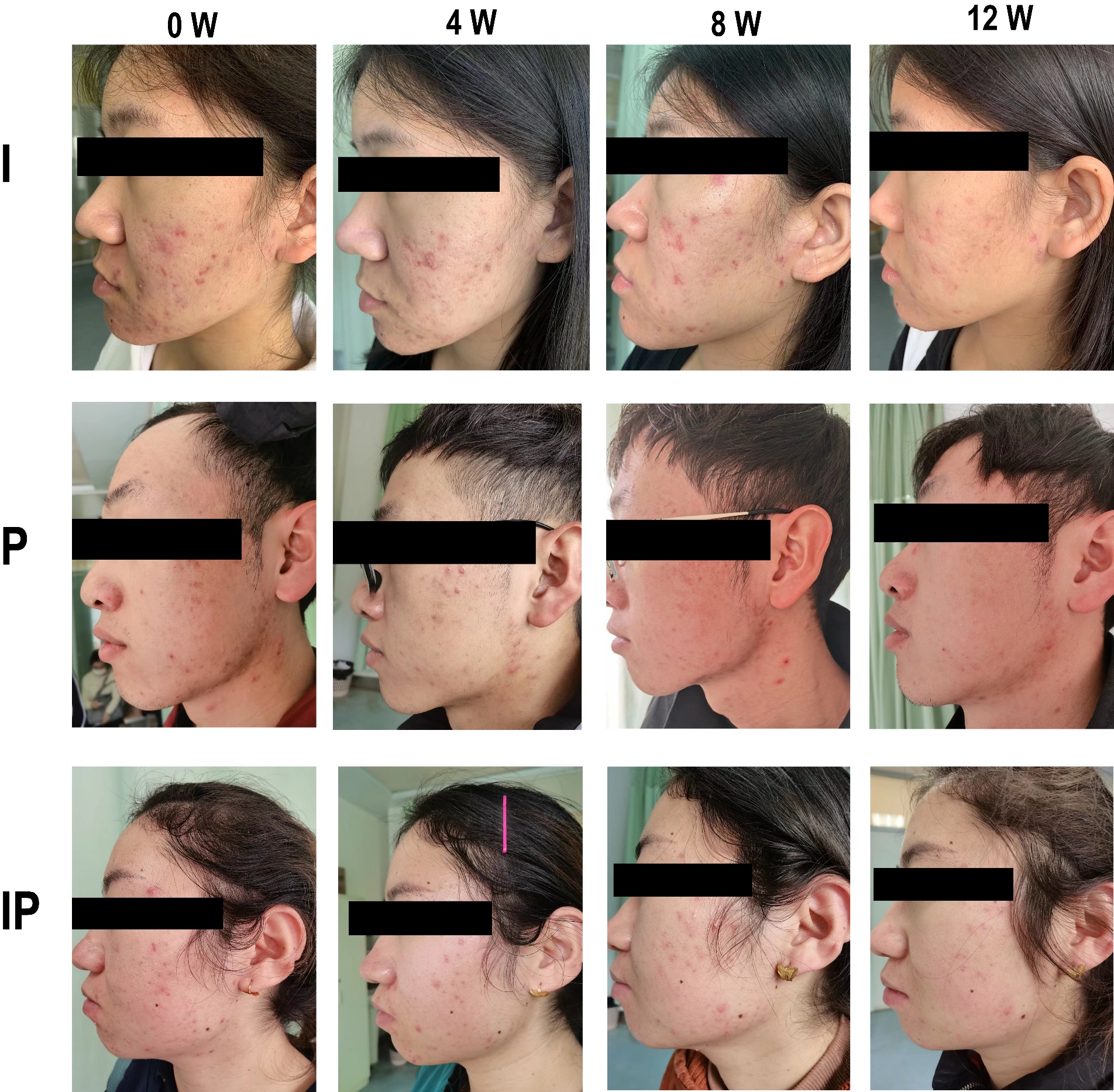


**Figure S1**. Oral isotretinoin and *L. plantarum* MH-301 improved symptoms in patients with acne vulgaris.
